# The Epoxidation of Limonene over the TS-1 and Ti-SBA-15 Catalysts

**DOI:** 10.3390/molecules191219907

**Published:** 2014-11-28

**Authors:** Agnieszka Wróblewska

**Affiliations:** Institute of Organic Chemical Technology, West Pomeranian University of Technology Szczecin, Pułaskiego 10, Szczecin 70-322, Poland; E-Mail: Agnieszka.Wroblewska@zut.edu.pl; Tel.: +48-91-449-4875; Fax: +48-91-449-4365

**Keywords:** limonene epoxidation, 1,2-epoxylimonene, TS-1, Ti-SBA-15, hydrogen peroxide, biomass

## Abstract

Limonene belongs to a group of very important intermediates used in the production of fine chemicals. This monoterpene compound can be obtained from peels of oranges or lemon which are a (biomass) waste from the orange juice industry. Thus, limonene is a renewable, easy available and a relatively cheap compound. This work presents preliminary studies on the process of limonene epoxidation over zeolite type catalysts such as: TS-1 and Ti-SBA-15. In these studies methanol was used as a solvent and as an oxidizing agent a 60 wt % hydrogen peroxide solution was applied. The activity of each catalyst was investigated for four chosen temperatures (0 °C, 40 °C, 80 °C and 120 °C). The reaction time was changed from 0.5 to 24 h. For each catalyst the most beneficial conditions (the appropriate temperature and the reaction time) have been established. The obtained results were compared and the most active catalyst was chosen. These studies have also shown different possible ways of limonene transformation, not only in the direction of 1,2-epoxylimonene and its corresponding diol, but also in direction of carveol, carvone and perillyl alcohol—compounds with a lot of applications. The possible mechanisms of formation of the allylic oxidation products were proposed.

## 1. Introduction

Limonene belongs to a group of very important intermediates for the production of fine chemicals. This monoterpene compound can be obtained from peels of oranges or lemon (biomass) which are a waste produced by the orange juice industry. The amount of limonene (*R*-enantiomer) obtained from peel oils reaches 90%–97%. Thus, limonene is a renewable, easy available and relatively cheap compound. It has been used in the production of cosmetics, secondary refrigerant fluids, paints, agrochemicals and in the cleaning industries [1,2,3]. The oxygenated derivatives of limonene, such as 1,2-epoxylimonene, 1,2-epoxylimonene diol, carvone, carveol and perillyl alcohol are very valuable intermediates used in the production of flavours, perfumes, cosmetics, food additives, drugs, agrochemicals [2,3,4] and polymers (for example “fragrant polymers”) [5,6]. Moreover, perillyl alcohol has been shown to be efficacious against the formation and progression of various cancers. It has shown therapeutic effects for pancreatic, mammary and liver tumors and chemopreventive ones for colon, skin and lung cancer [7,8].

It results from the literature review that the limonene epoxidation process is very complicated because apart from the main reaction—formation of 1,2-epoxylimonene ‒ secondary reactions can also occur in this process and formation of the following by-products is observed: 1,2-epoxylimonene diol, 8,9-epoxylimonene, 8,9-epoxylimonene diol, carvone, carveol, diepoxide (1,2 and 8,9), the diol of this epoxide, perillal, perillyl acid and perillyl alcohol [1,2,4,9,10,11,12]. The last of the mentioned by-products (perillyl alcohol) is usually formed in a trace amounts during the oxidation of limonene, and only in the presence of bacteria, fungus and yeasts was it a predominant product [13,14,15,16]. The possibility of the formation of allylic oxidation products by cyclic allylic hydrogen abstraction during oxidation of terpenes (3-carene and α-pinene—compounds with structures close to limonene) was also described by Rothenberg *et al.* [17]. In case of limonene epoxidation the allylic oxidation products are carveol—via cyclic allylic hydrogen abstraction (very often it undergoes further transformation to carvone) and perillyl alcohol—from acyclic allylic hydrogen abstraction.

Liquid phase oxidation of olefinic compounds by hydrogen peroxide or *t*-butyl hydroperoxide (TBHP) in the presence of heterogeneous catalysts is a very interesting way of obtaining very valuable, oxygenated products from olefins. A great advantage connected with the use of this kind of processes can be the ease of the separation and regeneration of heterogeneous catalysts and the possibility of obtaining interesting products with high selectivity. Several industrial processes have been developed on the basis of liquid phase oxidation, for example the epoxidation of propylene to propylene oxide. Among the heterogeneous catalysts special attention is directed to microporous and mesoporous titanium silicalite zeolite type catalysts, but there is a little information about the utilization of these catalysts in the epoxidation of limonene. Until now titanium silicalite catalysts such as Ti-MCM-41, Ti-MMM-2 and Ti-SBA-15 (only Ti-SBA-15 synthesized by a post-synthesis method) have been used in this process [9,11,18,19,20,21,22]. The studies on the epoxidation of limonene over the Ti-MCM-41 catalyst were mainly performed with help of hydrogen peroxide or TBHP as the oxidizing agents. Several methods were used for the synthesis of Ti-MCM-41 catalyst: direct—a sol-gel method under hydrothermal conditions (in-framework Ti-MCM-41) [9], and post-synthesis methods: grafting (Ti-grafted MCM-41) [18] or wetness and vet impregnation check nomenclature [20]. The the Ti-MCM-41 catalyst structure obtained in the direct sol-gel method was also silylated [19]. The oxidation studies over the various abovementioned Ti-MCM-41 catalysts were performed at temperatures of 70–85 °C, for a molar ratio of limonene/hydrogen peroxide = 3.7:1 and for the reaction time of 0.5–7 h (in one case from 1 h to 24 h [18]). Over the direct synthesised Ti-MCM-41 catalyst the selectivity of the epoxide compounds (the sum of 1,2- and 8,9-epoxylimonene) was about 60 mol %. Carveol and carvone were formed with a selectivity of 20 mol %, diepoxylimonene with a selectivity of about 10 mol % and glycols about 10 mol %. The conversion of limonene ranged from 36 to 80 mol %. Over the silylated Ti-MCM-41 catalyst the results of limonene epoxidation were very close, and the only significant difference was a higher value of the hydrogen peroxide efficiency [19]. The two other methods of Ti-MCM-41 preparation (wetness and vet impregnation) do not cause important changes in the catalyst activity, and only a slight decrease in the epoxide selectivity was observed [20]. The utilization of TBHP in the epoxidation of limonene leads to a 62 mol % conversion of limonene and a and 75 mol % selectivity for 1,2-epoxylimonene after a reaction time of 24 h. Epoxidation of limonene over the Ti-MMM-2 catalyst was performed in acetonitrile as the solvent, at a temperature of 60 °C and with a limonene/H_2_O_2_ molar ratio of 1:2 [11]. These studies showed that the amount of the epoxide compound obtained was three times higher than that of the other products. Simultaneously, the amount of carvone was higher than that of carveol. Moreover, in the post-reaction mixtures perillyl alcohol was also detected [11]. During the studies over the Ti-SBA-15 catalyst synthesized by grafting titanium on a SBA-15 structure two oxidizing agents were used: hydrogen peroxide and TBHP [21,22]. The reaction was performed in acetonitrile as the solvent and for a reaction time up to 24 h. The following molar ratios of limonene/oxidizing agent were used in these studies: the molar ratio of limonene/H_2_O_2_ = 1:1.3 and the molar ratio of limonene/TBHP = 1:1.6. For the reaction with H_2_O_2_ the temperature of 70 °C and for TBHP the temperature of 80 °C were used. For all investigated oxidizing agents the 1,2-epoxylimonene selectivity was 100 mol %, but the conversion of limonene was 40 mol % for H_2_O_2_, and 60 mol % for TBHP. 

The aim of this work was to study limonene epoxidation over two titanium silicalite zeolite type catalysts: microporous (TS-1) and mesoporous (Ti-SBA-15). The Ti-SBA-15 catalyst used in these studies was synthesized by a direct hydrothermal method (the Ti-SBA-15 catalyst synthesized by this method has not been previously used in the epoxidation of limonene). Moreover, it results from our previous studies that the Ti-SBA-15 catalyst is more stable under the reaction conditions during the epoxidation than the Ti-MCM-41 catalyst (also a mesoporous material), taking into account the structures of these materials [23,24]. Broad studies on limonene epoxidation over the TS-1 catalyst have also not been performed. It will be very interesting to compare the activity of the TS-1 and the Ti-SBA-15 catalysts in the process of limonene epoxidation. The studies were performed at four chosen temperatures and for reaction times from 0.5 h to 24 h. In the limonene epoxidation methanol was used as the solvent and 60 wt % hydrogen peroxide as the oxidizing agent. For each catalyst the most beneficial conditions (temperature and the reaction time) have been established. The obtained results were compared and the most active catalyst was chosen. The possibility of the obtaining of various products in this process was shown and possible mechanisms were proposed for formation of the allylic oxidation products. 

## 2. Results and Discussion

The studies on the influence of the temperature and the reaction time on the course of limonene epoxidation over the TS-1 and Ti-SBA-15 catalysts are presented in Table 1 (for the TS-1 catalyst) and Table 2 (for the Ti-SBA-15 catalyst). 

**Table 1:** Studies on the influence of the temperature and the reaction time on the course of limonene epoxidation over the TS-1 catalyst.

	Reaction Time [h]						
	0.5	1.0	1.5	2.0	2.5	3.0	24
**Temperature 0 °C**							
**S_1,2-EL_**	91	91	91	93	92	92	84
**S_diol of 1,2-EL_**	9	9	9	7	8	8	14
**C_limonene_**	2	2	2	2	2	2	2
**S_org.comp./H_2_O_2__**	3	2	2	3	3	3	3
**Temperature 40 °C**							
**S_1,2-EL_**	50	42	34	32	29	29	10
**S_diol of 1,2-EL_**	13	14	11	11	12	13	15
**S_carveol_**	0	2	5	5	8	5	5
**S_perillyl alcohol_**	37	42	50	52	51	53	70
**C_limonene_**	2	3	4	4	5	5	6
**S_org.comp./H_2_O_2__**	3	4	5	5	6	6	8
**Temperature 80 °C**							
**S_1,2-EL_**	34	28	25	23	23	20	11
**S_diol of 1,2-EL_**	12	11	11	11	11	11	11
**S_carvone_**	18	15	16	16	15	16	17
**S_carveol_**	0	0	1	2	4	5	11
**S_perillyl alcohol_**	64	46	47	48	47	48	50
**C_limonene_**	4	5	5	6	7	7	24
**S_org.comp./H_2_O_2__**	6	7	8	9	10	12	33
**Temperature 120 °C**							
**S_1,2-EL_**	26	22	22	22	21	19	0
**S_diol of 1,2-EL_**	8	11	11	11	11	12	27
**S_carvone_**	12	16	17	17	17	17	19
**S_carveol_**	0	0	2	4	5	6	8
**S_perillyl alcohol_**	34	51	52	46	46	46	46
**C_limonene_**	5	5	8	8	10	10	45
**S_org.comp./H_2_O_2__**	8	8	11	12	15	15	59

EL—epoxylimonene, S—selectivities of the appropriate products, C—conversion, S_org.comp./H_2_O_2__ selectivity of transformation to organic compounds in relation to hydrogen peroxide consumed

**Table 2:** Studies on the influence of the temperature and the reaction time on the course of limonene epoxidation over the Ti-SBA-15 catalyst.

	Reaction Time [h]						
	0.5	1.0	1.5	2.0	2.5	3.0	24
**Temperature 0 °C**							
**S_1,2-EL_**	68	57	28	11	11	4	4
**S_diol of 1,2-EL_**	6	7	10	12	12	13	13
**S_carvone_**	5	10	19	13	13	9	8
**S_carveol_**	0	0	0	0	0	3	5
**S_perillyl alcohol_**	21	26	43	64	64	71	70
**C_limonene_**	5	5	6	6	6	18	18
**S_org.comp./H_2_O_2__**	8	9	10	10	10	27	27
**Temperature 40 °C**							
**S_1,2-EL_**	48	29	13	9	6	3	3
**S_diol of 1,2-EL_**	6	7	10	10	11	11	11
**S_carvone_**	13	14	14	14	14	16	16
**S_carveol_**	1	4	4	5	5	5	5
**S_perillyl alcohol_**	32	46	59	62	64	65	65
**C_limonene_**	9	13	15	21	22	37	40
**S_org.comp./H_2_O_2__**	19	25	30	40	40	62	54
**Temperature 80 °C**							
**S_1,2-EL_**	26	15	8	6	5	4	3
**S_diol of 1,2-EL_**	8	10	11	11	11	13	13
**S_carvone_**	8	12	12	13	15	16	17
**S_carveol_**	2	2	2	2	2	2	3
**S_perillyl alcohol_**	56	61	67	68	67	65	64
**C_limonene_**	17	22	29	33	36	42	46
**S_org. comp./H2O2_**	36	43	52	52	54	59	64
**Temperature 120 °C**							
**S_1,2-EL_**	20	11	5	5	3	2	0
**S_diol of 1,2-EL_**	10	10	11	11	12	12	12
**S_carvone_**	12	14	16	16	17	17	19
**S_carveol_**	2	2	3	3	3	4	4
**S_perillyl alcohol_**	56	63	65	65	65	65	65
**C_limonene_**	23	29	32	35	40	52	52
**S_org.comp./H_2_O_2__**	30	34	37	41	46	59	57

EL—epoxylimonene, S—selectivities of the appropriate products, C—conversion, S_org.comp./H_2_O_2__ selectivity of transformation to organic compounds in relation to hydrogen peroxide consumed.

**Scheme 1:**
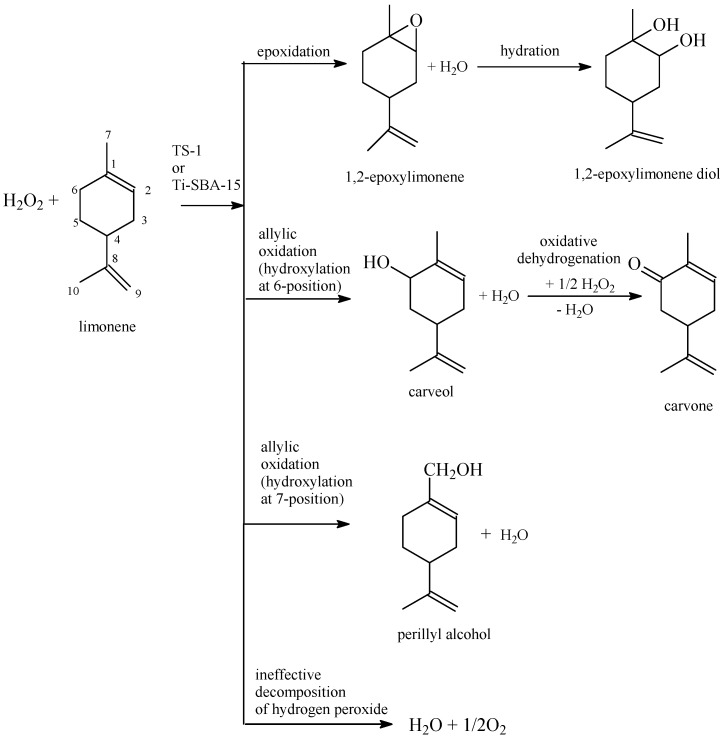
Reactions during the process of limonene epoxidation over the TS-1 and the Ti-SBA-15 catalysts.

The results presented in Table 1 and Table 2 show that the process of limonene epoxidation is very complicated and can be generally described as shown in Scheme 1.

In this process, as a result of the electrophilic attack on the double bond (position 1–2) in the limonene molecule 1,2-epoxylimonene is formed. 1,2-Epoxylimone can undergo further hydration of the epoxide ring, and formation of 1,2-epoxylimonene diol is observed. The next product of this process is carveol. This compound is obtained as a result of the cyclic allylic hydrogen abstraction (allylic oxidation-hydroxylation at the 6-position [1,13,17]). As a result of the acyclic allylic hydrogen abstraction (allylic oxidation-hydroxylation at the 7-position [1,13,17]) perillyl alcohol is formed. Among the products of this process carvone is also observed. This compound is formed as a result of oxidative dehydrogenation of carveol [25]. In the case of allylic oxidation at the 7-position the product of perillyl alcohol oxidative dehydrogenation (perillyl aldehyde) was not observed. For this process the phenomenon of an ineffective decomposition of hydrogen peroxide which can happen very fast under the studied conditions, especially at raised temperatures, is also very important. This phenomenon is characteristic of oxidation processes which use hydrogen peroxide as the oxidizing agent and which are performed in the presence of solid acids and has been described by various authors [26,27,28]. Therefore, for the description of this process a very important function was introduced—the selectivity of transformation to organic compounds in relation to hydrogen peroxide consumed (Table 1 and Table 2). This function describes how much of the reacted hydrogen peroxide is converted effectively and takes part in the oxidation of organic compounds. The difference between 100 mol % and value of the above function shows how much of the reacted hydrogen peroxide undergoes ineffective decomposition.

Studies on the epoxidation of limonene over the TS-1 catalyst (Table 1) and at the temperature of 0 °C show that it is possible to obtain 1,2-epoxylimonene with a very high selectivity (above the value of 90 mol %), when the epoxidation process is carried out for the reaction time of 0.5–3 h, but these high values of the selectivity of the epoxide are obtained at a low conversion of limonene—2 mol %. As only by-product in this process 1,2-epoxylimonene diol is formed with a selectivity of 9–14 mol %. The values of the selectivity of 1,2-epoxylimonene diol are not high and this shows that the epoxide compound is stable under these conditions. The function of the selectivity of transformation to organic compounds in relation to hydrogen peroxide consumed takes very low values independent of the reaction time and amounts to 2–3 mol %. It shows that almost 97% of the reacted hydrogen peroxide undergoes ineffective decomposition. 

At the temperature of 40 °C, apart from 1,2-epoxylimonene and its diol, carveol and perillyl alcohol are also detected in the reaction mixture, but the product of the hydroxylation at the 7 position is predominant. Perillyl alcohol is formed with a selectivity of 37 mol % (reaction time of 0.5 h) to 70 mol % (reaction time of 24 h). The selectivity of 1,2-epoxylimonene at the temperature of 40 °C is lower than the value of this function obtained for the temperature of 0 °C, even for the very short reaction time—0.5 h (50 mol %). The same as for the temperature of 0 °C, a part of this epoxide compound undergoes hydration to diol (the diol selectivity amounts to 11–15 mol %). For the temperature of 40 °C the conversion of limonene is a little higher than for the temperature of 0 °C and amounts to 6 mol %. The selectivity of transformation to organic compounds in relation to hydrogen peroxide consumed takes values of about 2–6 mol %. This shows that the hydrogen peroxide undergoes about 94%–98% ineffective decomposition, in a very similar result as seen for the temperature of 0 °C. 

For the temperature of 80 °C the selectivity of 1,2-epoxylimonene is lower than for the temperatures of 0 °C and 40 °C, and amounts to 34–11 mol % for the reaction time of 0.5–24 h. A part of this epoxide undergoes hydration to diol (the diol selectivity amounts to 11–12 mol %, independent of the reaction time). In the reaction mixture not only the epoxide compound, its diol, carveol and perillyl alcohol were detected, but also carvone. The formation of this last compound was not observed at the temperatures of 0 °C and 40 °C. At the temperature of 80 °C and for the reaction time 0.5–1.0 h the totality of the carveol formed is converted to carvone. For the longer reaction time carveol is detected in the reaction mixture, but its selectivity is very low—1–11 mol %—but most of this compound still undergoes conversion to carvone. At the temperature of 80 °C the predominant product is perillyl alcohol, which is formed in the studied range of reaction times with a selectivity ranging from 64 mol % to 50 mol %. For this temperature the limonene conversion is slightly higher and amounts to from 4 mol % to 24 mol % for the reaction time from 0.5 to 24 h. Moreover, the selectivity of transformation to organic compounds in relation to hydrogen peroxide consumed changes significantly as the reaction time is extended from 6 mol % (0.5 h) to 33 mol % (24 h). Such values of this function were not observed for the lower studied temperatures. 

For the temperature of 120 °C, like the temperature of 80 °C, during the process of the epoxidation of 1,2-epoxylimone, its diol, carveol, carvone and perillyl alcohol are formed. A part of the 1,2-epoxylimonene is hydrolyzed to diol for the reaction time from 0.5 to 3 h, and for the reaction time of 24 h the whole amount of epoxide is converted to diol. A large part of the carveol is also oxidized to carvone (for the reactions time of 0.5 and 1 h all the carveol is converted to carvone). The conversion of limonene at the temperature of 120 °C is higher than for the temperature of 80 °C, especially for the reaction time of 24 h. The selectivity of transformation to organic compounds in relation to hydrogen peroxide consumed changed significantly as the reaction time was extended from 8 mol % (0.5 h) to 59 mol % (24 h). The obtained values of this function are higher than for the temperature of 80 °C. 

**Scheme 2:**
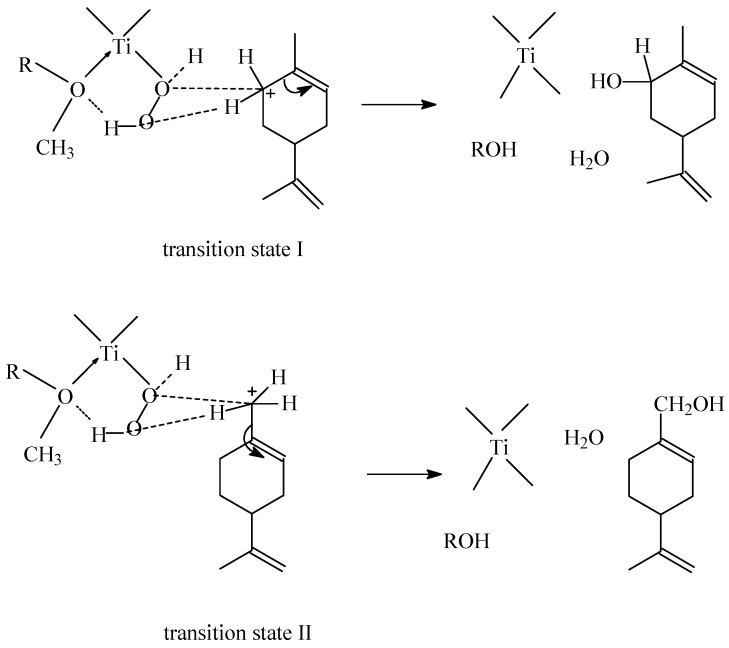
The mechanism of allylic oxidation product formation (carveol and perillyl alcohol) over the TS-1 catalyst.

Studies on the epoxidation of limonene over the TS-1 catalyst show that generally at higher temperatures (40 °C, 80 °C and 120 °C) allylic oxidation products predominate over other products. The first of this kind of products is carveol which is obtained as a result of cyclic allylic hydrogen abstraction. The second product is perillyl alcohol which is obtained as a result of acyclic allylic hydrogen abstraction. Rothenberg *et al.* [17] studied the epoxidation of 3-carene and α-pinene (two compounds with the structures close to that of the limonene molecule) by dioxygen in the presence of various homogenic catalysts and they showed that in case of these two compounds the so-colled “cyclic activation” factor—a result of appropriate transition state formation by overlapping of orbitals, is a main cause of the formation of allylic oxidation products by cyclic allylic hydrogen abstraction. Essential for this reaction is the skeletal flexibility of the organic compound molecule and formation of a boat conformation. In case of the studies presented in this work, the dominant product is the acyclic allylic hydrogen abstraction product—perillyl alcohol. An explanation of this direction of the reaction over the TS-1 catalyst can be proposed on the basis of the mechanism of allylic oxidation product formation (carveol and perillyl alcohol) (Scheme 2).

According to the mechanism presented above the formation of allylic oxidation products can be a result of a shift of electrons in the direction of the double bond in limonene (from position 6 or 7) and creation of a positive charge on the 6 or 7 C atom, and simultaneously the interaction between the active 5-membered complex of Ti and the atom of C (position 6 or 7). During this interaction water molecule is formed and an –OH group is transferred to the limonene molecule at the C atom 6 or 7 in limonene. 

**Figure 1:**
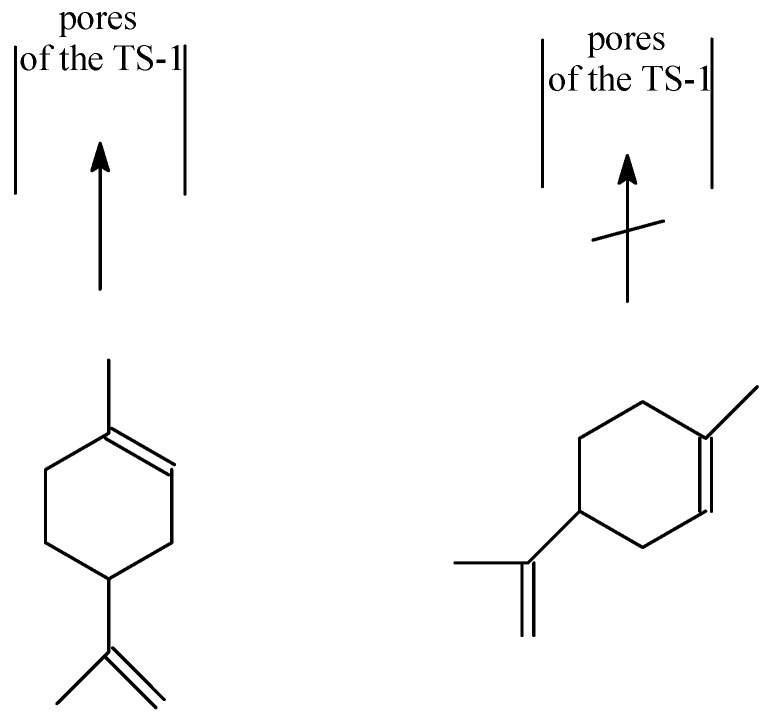
The possible mode of entry of the limonene molecules into the narrow pores of the TS-1 catalyst.

Taking into account the transition states I and II presented above, the following factors should be taken into consideration in order to explain the course of the studied process: an ease of formation of these transition states (amount of the energy which is needed for the creation of these transition states), their size and the steric hindrance in the space around these transition states. The domination of perillyl alcohol in relation to carveol in the post-reaction mixtures shows that the transition state II is formed more preferably than the transition state I. The steric limitations connected with the access to the C atom at position 6 in comparison to C atom at position 7 are probably the main reason for this situation. The second cause of the preferred formation of perillyl alcohol in comparison to carveol can be the differences in the place where this transition state can be created. Probably in case of the TS-1 catalyst the transition state I is preferably created on the external surface of the catalyst because it needs more space and the transition state II is created inside the pores of this catalyst. These conclusions can be explained on the basis of the studies performed by Rothenberg *et al.* [17]. These authors shows that the terpene rings with a structure similar to limonene have the ability to change conformation because of the flexibility of this ring. It is possible that in the case of perillyl alcohol formation the ring takes such conformation which causes that the width of the ring to be so small that this compound can enter into the narrow pores of the TS-1 catalyst. Thus can be generally illustrated as shown in Figure 1.

The situation where one product is formed inside the pores and the second one on the external surface of the catalyst has already been described for the process of phenol hydroxylation over titanium silicalite catalysts [28,29]. 

**Figure 2:**
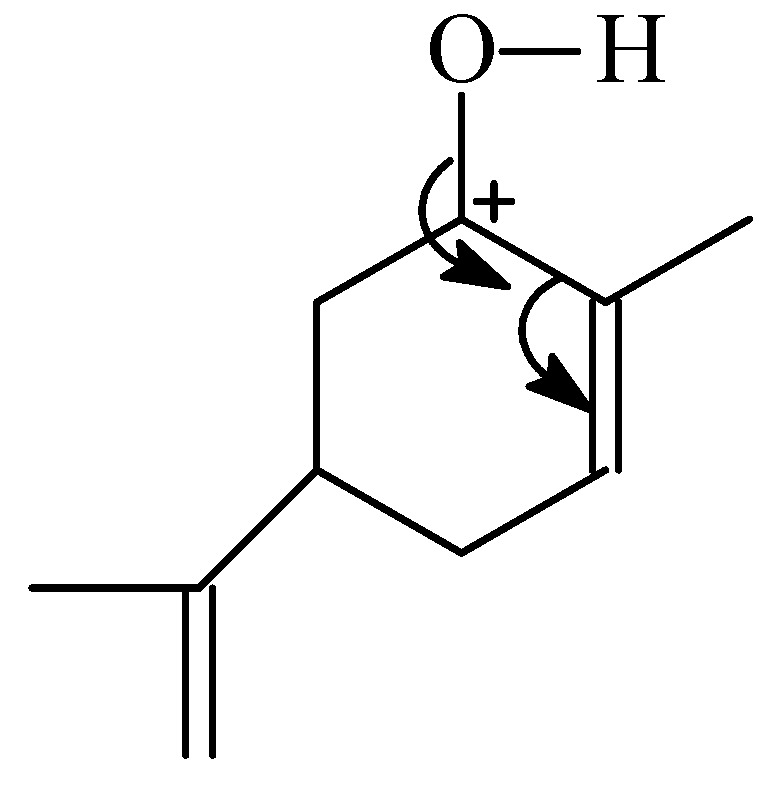
The structure which is formed during the oxidative dehydrogenation of carveol to carvone.

The possibility of the very easy formation of carvone from carveol over the titanium silicalite TS-1 catalyst can be also explained on the basis of the mechanism presented above. In case of perillyl alcohol which is formed inside the pores the formation of transition state which is needed for oxidative dehydrogenation is difficult because a lack of the space for this transition state. In the case of carveol which is probably formed on the external surface of the TS-1 catalyst, the spatial hindrance does not occur and thus the conversion of carveol to carvone can go very easily. Apart from the site of carveol formation and the steric limitation influence, there is a third factor that can also have an influence on the easiness of carvone formation—the place in the limonene molecule at which the oxidative dehydrogenation takes place. The ring probably influences this reaction by shifting the electrons of the ring and formation of a positive charge at the C6 atom of the carveol molecule. The structure which can be obtained in this way is presented below (Figure 2).

**Figure 3:**
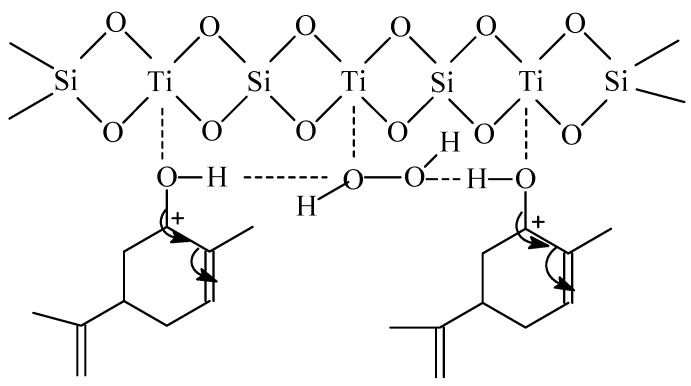
The mechanism of carvone formation over the titanium silicalite catalyst.

The formation of this structure causes the H atom bonded with the oxygen atom in the –OH group to be easily abstracted. The formation of carvone from carveol on the active site of Ti (on the external surface of the catalyst but also on the active sites inside the pores—this last situation will occur in the next part of this work for studies on the Ti-SBA-15 catalyst) can be explained on the basis of the studies presented by Ramos-Fernandez *et al.* [25] and performed for titania mesoporous material. Taking into account these results the mechanism of carvone formation over the titanium silicalite catalyst shown in Figure 3 can be proposed.

The studies on the epoxidation of limonene over the TS-1 catalyst also show that the effective utilization of hydrogen peroxide, which is shown as a function of the selectivity of transformation to organic compounds in relation to hydrogen peroxide consumed, rises as the temperature of the epoxidation rises and the reaction time is prolonged. The highest value of this function is obtained for the temperature of 120 °C and for the reaction time of 24 h (59 mol %). This shows that at lower temperatures the process ineffective decomposition of the hydrogen peroxide occurs predominantly and the transition states connected with the oxidation processes of limonene molecules are not easily formed. On the other hand, the temperature increase causes the transition states connected with the oxidation processes of limonene molecules to be formed more easily but still more than half of the hydrogen peroxide if converted ineffectively. Thus, the utilization of an excess of hydrogen peroxide in these studies is justified—the molar ratio of limonene/H_2_O_2_ used for these studies amounts to 1:2. 

The studies over the mesoporous titanium silicate catalyst Ti-SBA-15 (Table 2) and at the temperature of 0 °C show that at this very low temperature it is impossible to obtain the very high 1,2-epoxylimonene selectivity. The highest selectivity for this compound, which was obtained for the reaction time of 0.5 h, amounts to 68 mol %. For the reaction time from 1.5–2 h about half of the amount of epoxide compound undergoes hydration to diol, and for longer reaction times (from 3 h to 24 h) almost all of the 1,2-epoxylimonene undergoes hydration. In this temperature carveol, carvone and perillyl alcohol are also formed. Simultaneously, almost all the obtained carveol is converted to carvone. For the reaction time from 1.5 to 24 h perillyl alcohol is the main product of the process (its selectivity amounts to 43 to 70–71 mol %). The conversion of limonene changed from 5 to 18 mol %. For this very low temperature the selectivity of transformation to organic compounds in relation to hydrogen peroxide consumed changes from 8 mol % to 27 mol % and it is higher than for the TS-1 catalyst. 

The raising of the temperature of the epoxidation process over the Ti-SBA-15 catalyst to 40 °C causes the selectivity for the epoxide compound for the reaction time of 0.5 h to be lower than for the temperature of 0 °C and amount to 48 mol %. On the other hand, for this reaction time carvone and perillyl alcohol are formed with higher selectivities: 13 and 32 mol %, respectively. The conversion of limonene is considerably higher at this temperature and increases from 19 to 40 mol % as the reaction time is prolonged. The selectivity of transformation to organic compounds in relation to hydrogen peroxide consumed also rises considerably for the temperature of 40 °C in comparison to the temperature of 0 °C, and amounts from 19 to 73 mol %.

For the studies over the Ti-SBA-15 catalyst at higher temperatures—80 and 120 °C—the epoxide compound selectivity for the reaction time of 0.5 h decreases to 26 and 20 mol %, respectively. For longer reaction times, almost the whole amount of the epoxide compound undergoes hydration to diol just as almost the whole amount of carveol undergoes conversion to carvone. The perillyl alcohol selectivity is practically unchanged with changing reaction time and amounts to about 56‒65 mol % for the temperatures of 80 °C and 120 °C. The conversion of limonene and the selectivity of transformation to organic compounds in relation to hydrogen peroxide consumed are also similar for these two temperatures during the changes of the reaction time and amount to 17‒52 mol % and 32‒64 mol %. 

The comparison the results obtained over the mesoporous Ti-SBA-15 catalyst shows that independent of the temperature of the process 1,2-epoxylimonene, its diol, carveol which very easily undergoes oxidative dehydrogenation to carvone and perillyl alcohol which is very stable under the reaction conditions and does not react to perillyl aldehyde are present in the post-reaction mixture. The studies also show that perillyl alcohol is the main product of this process. Taking into account the size of the pores of the Ti-SBA-15 material (5.0 nm) probably all of these products can be formed inside the pores and also outside the pores on the surface, but the main factor which should be considered for the explanation the preferable formation of perillyl alcohol over the Ti-SBA-15 catalyst is the ease of formation of the transition state I and transition state II at the active centers of Ti which were presented during the explanation the results for the TS-1 catalyst. Probably, the transition state II is an active derivative which needs lees energy for its formation and this formation is connected with lower steric limitations, thus the perillyl alcohol is the main product, independent of where this reaction takes place. 

The studies on the epoxidation of limonene over the Ti-SBA-15 catalyst show, like for the TS-1 catalyst, that the effective utilization of hydrogen peroxide increases as the temperature of the epoxidation increases and as the reaction time is prolonged, but the highest values of this function are not only obtained for the temperature of 120 °C but also for lower temperatures (40 °C and 80 °C) and for a shorter reaction time of 3‒24 h (54‒64 mol %) than for the TS-1 catalyst. These results show that over this catalyst the reactions connected with oxidation processes and oxidative dehydrogenation process take place faster than over the TS-1 catalyst and this is the result of the size of the pores of this catalyst, the developed specific surface area and hence better accessibility to the active Ti centers. 

## 3. Experimental Section 

The TS-1 catalyst was prepared according to the method described by Thangaraj *et al.* [30]. The molar ratio of Si/Ti in the gel before crystallization was 64:1. The Ti content in this catalyst was 3.01 wt %, the specific surface area amounted to 372 m^2^/g, the size of the pores achieved 0.5 nm and the crystal size amounted to 0.5 μm. The full characterization of this catalyst was presented in our previous article [31]. The Ti-SBA-15 was synthesized by the method of Berube *et al.* [32]. The molar ratio of Si/Ti in the gel before crystallization was 40:1. The Ti content in this catalyst was 2.46 wt %, the specific surface area amounted to 622 m^2^/g, the size of the pores achieved 5.0 nm and the particles of this catalyst had a shape of the rods with the width of about 3‒4 μm and the length of 15 μm and were composed with smaller particles with the width of 0.54 μm and the length of 0.76 μm. The full characteristics of the Ti-SBA-15 catalyst were presented in our previous article [33]. 

In the epoxidation of limonene the following raw materials were used: *R*-(+)-limonene (97%, Sigma, St. Louis, MO, USA), hydrogen peroxide (60 wt % water solution, Chempur, city, state abbrev of US, country), and methanol (analytical grade, Chempur). The studies over the TS-1 and the Ti-SBA-15 catalysts were performed for four temperatures: 0 °C, 40 °C, 80 °C and 120 °C. The reaction times ranged from 0.5 h to 24 h. The other parameters were as follows: the molar ratio of limonene/H_2_O_2_ = 1:2, solvent (methanol) concentration of 80 wt % and catalyst content of 3 wt %. The process was carried out in a glass reactor with a capacity of 25 cm^3^ equipped with a reflux condenser, a thermometer and a magnetic stirrer. The raw materials were introduced into the reactor in the following order: catalyst, limonene, methanol and 60 wt % aqueous solution of hydrogen peroxide. The temperature was achieved with help of a silicone oil bath. The progress of the reaction was studied after the following reaction times: 0.5 h, 1 h, 1.5 h, 2 h, 2.5 h, 3 h and 24 h. Samples at different reaction times were analysed by a GC-method on a Focus apparatus equipped with a flame-ionization detector and fitted with the Restek Rtx-WAX capillary column filled with polyethylene glycol. The parameters of the analyses were as follows: helium pressure of 50 kPa, sensitivity of 100, the temperature of the sample chamber 200 °C, the detector temperature 250 °C, the temperature of the thermostat was increased according to the following program: isothermally at 60 °C for 2 min, an increase to 240 °C at the rate of 15 °C/min, isothermally at 240 °C for 4 min, cooling to 60 °C. Reaction products were also identified by GC-MS. The hydrogen peroxide conversion was measured by iodometric titration. The mass balance for each sample taken for the appropriate reaction time was calculated. On the basis of the mass balance the main functions describing the process were determined: the selectivities of the appropriate products, conversion of limonene, conversion of hydrogen peroxide and the selectivity of transformation to organic compounds in relation to hydrogen peroxide consumed (efficiency of hydrogen peroxide conversion). 

## 4. Conclusions 

Studies on the epoxidation of limonene performed over the microporous TS-1 catalyst and the mesoporous Ti-SBA-15 catalyst showed a lot of differences in the presence of these catalysts taking into account not only the values of the main functions describing the process, but also the place on the surface of the catalysts at which the main reactions characterizing this process can proceed. The microporous TS-1 catalyst is an active catalyst in the process of limonene epoxidation but higher values of the main functions describing the process were obtained for the mesoporous Ti-SBA-15 catalyst. Especially, a low activity of the TS-1 catalyst is evident at lower values of the conversion of limonene and the selectivity of transformation to organic compounds in relation to hydrogen peroxide consumed (effective conversion of hydrogen peroxide). 

For both the studied catalysts the formation of carveol is observed and also for both of these catalysts almost whole amount of the carveol obtained is converted to carvone independent of the temperature and the reaction time. The amount of carveol and carvone rises with the increasing temperature of the reaction and with the extension of the reaction time. This shows that higher temperatures are more beneficial for obtaining this allylic oxidation reaction product. This conclusion is also true for perillyl alcohol which is also formed as an allylic oxidation product and for almost all studied conditions is the main product of the process. The obtained perillyl alcohol is stable under the conditions in which the epoxidation was performed and its conversion to perillyl aldehyde was not observed. For the allylic oxidation products (carveol and perillyl alcohol) possible transitions states (I and II) can be proposed. For the studies over the TS-1 catalyst where to explain the obtained results the size of the pores should also be taken into consideration, two different sites of formation of allylic oxidation products can be proposed. Taking into account the size of the limonene molecule, the size of the pores and the structure of the transition states I and II, for the formation of perillyl alcohol it is proposed that this reaction probably proceeds inside the narrow pores of the TS-1 catalyst. This is possible because the ring of the limonene molecule is flexible and probably it can adopt a configuration in which this ring is very narrow and can enter the pores. On the other hand, the narrow size of the pores ensures that further transformation of perillyl alcohol into the corresponding aldehyde does not occur. For the formation of carveol and its further transformation to carvone it was proposed that these reactions take place on the outside surface of the TS-1 catalyst. The active centers on the outside surface are more preferable, because of the steric hindrance connected with the size of the transition state I. The easy conversion of carveol to carvone over the TS-1 and the Ti-SBA-15 catalyst in comparison to perillyl alcohol was explained on the basis of the transition state in which a main role is played by the ring and its stabilizing effect. In the case of the perillyl alcohol oxidative dehydrogenation this effect does not exist because the –OH group is not directly connected with the ring.

It was assumed for the studies over the Ti-SBA-15 that the reactions of allylic oxidation and oxidative dehydrogenation can happen inside the wide pores of this catalyst and also on the outside surface of this catalyst. These studies also show that during the allylic oxidation the formation of the transition state II is preferred in comparison to transition state I—perillyl alcohol was also the main product during the process performed over the Ti-SBA-15 catalyst. Probably, the transition state II needs less energy for formation and this formation is connected with lower steric limitations. 

The selectivities of transformation to organic compounds for the Ti-SBA-15 catalyst are considerably higher, independent of the temperature of the process. Also for shorter reaction times hydrogen peroxide is used more effectively in the oxidation reactions than for the TS-1 catalyst. Probably, an easier access of limonene molecules to the active centers of Ti and thus the easier formation of the transition states of various types depending on the reaction (epoxidation, allylic oxidation, oxidative dehydrogenation) play an important role in this process and control the effective transformation of hydrogen peroxide. 
